# Usnic acid impacts energy production and iron metabolism in *Mycobacterium tuberculosis* H37Rv

**DOI:** 10.1128/msystems.00256-25

**Published:** 2025-04-09

**Authors:** Rafał Sawicki, Anna Zabost, Grzegorz Jankowski, Ewa Augustynowicz-Kopeć, Wiesław Truszkiewicz, Elwira Sieniawska

**Affiliations:** 1 Department of Biochemistry and Biotechnology, Medical University of Lublin 49554, Lublin, Poland; 2 Department of Microbiology, National Tuberculosis and Lung Diseases Research Institute 49608 , Warsaw, Poland; 3 Department of Natural Products Chemistry, Medical University of Lublin 49554, Lublin, Poland; University of California San Diego, La Jolla, California, USA

**Keywords:** tuberculosis, metabolomics, lipidomics, transcriptomics, natural products, antimicrobials, mechanism of action, iron metabolism, mycobactins

## Abstract

*Mycobacterium tuberculosis* has developed a wide array of response mechanisms to various stress factors. Usnic acid has been demonstrated to be a potent antimycobacterial agent that induces stress responses and growth inhibition in many mycobacterial species. Previous studies have shown that it alters the expression of stress-responsive sigma factors, as well as the metabolites and lipid profile in *M. tuberculosis* H37Ra. This study was designed to examine potential differences in the strain-specific susceptibility of the virulent H37Rv strain to usnic acid. By combining lipidomic and transcriptomic analyses, we uncovered the impact of usnic acid on bacterial metabolism. The observed downregulation of key lipid classes suggested reduced metabolic activity. The simultaneous elevation of mycobactins—siderophores used by members of the genus *Mycobacterium* to transport free extracellular iron ions into the cytoplasm—indicated the involvement of iron in the stress response generated by usnic acid. The repressed tricarboxylic acid (TCA) cycle and oxidative phosphorylation were compensated by the upregulation of alternative energy production pathways, such as cytochrome P450 and the ferredoxin reductase system. This indicates that mycobacteria may switch to alternative electron transport mechanisms under usnic acid stress using iron-sulfur clusters to generate energy. From a therapeutic perspective, the study highlights iron metabolism as an essential drug target in mycobacteria. Simultaneously, the results confirm the strain-specific metabolic response of sister strains against the same stressing agent.

**IMPORTANCE:**

A previous study on the influence of usnic acid on the avirulent H37Ra strain revealed that the early bacterial response was associated with redox homeostasis, lipid synthesis, and nucleic acid repair. The response of bacteria to antimicrobials is specific to each species and strain. Given the genetic and phenotypic differences between the avirulent H37Ra strain and the virulent H37Rv strain, we combined lipidomics and global transcriptomics to uncover the mechanism of action of usnic acid against H37Rv. The study identified strain-specific differences between the virulent H37Rv and avirulent H37Ra. The H37Ra strain exhibited increased metabolic activity, while the H37Rv strain showed a reduction in basic metabolic processes and activated alternative iron-dependent energy production. These differences highlight the varying susceptibility of sister strains within the same species to the same antibacterial agent.

## INTRODUCTION

Because tuberculosis is still a threatening disease (1), research on new antimycobacterial compounds or new drug targets in mycobacterial cells is necessary. The rise in drug-resistant strains, such as multi-drug resistant and extensively-drug resistant *Mycobacterium tuberculosis*, creates an urgent need for innovative treatments. New drugs should target novel molecular pathways and overcome resistance mechanisms, providing effective treatment options. Additionally, current therapies are often lengthy, costly, and accompanied by significant side effects (2). New drugs should have the potential to shorten treatment durations and improve patient comfort. More effective therapies could also help combat tuberculosis more efficiently, especially in low-income regions where access to treatment is limited. Furthermore, innovative drugs may have potential applications in treating other infectious diseases, offering additional benefits to public health by exploring new mechanisms of action and enhancing the chances of successful treatment.

*M. tuberculosis* complex (MTBC) has been widespread in the environment, including soil, water, pasture, air, and dust. This environmental contamination of MTBC is implicated in indirect tuberculosis transmission to humans and animals (3). Considering this fact, it should be noted that nature produces compounds preventing the spreading of contamination mentioned above. Lichens exemplify an ecologically obligate, stable mutualism between a fungal partner (the mycobiont) and an inhabitant population of extracellularly located unicellular or filamentous algal or cyanobacterial cells (the photobiont) (4). Lichens serve as a food source and habitat for many animals such as deer, birds, and rodents (5). The lichen species, *Cetraria islandica*, has been used in tuberculosis in several countries including Spain, France, and Turkey. Its antimycobacterial properties have been attributed mainly to lichen acids (fumaroprotocetraric, protocetraric, protolichesterinic, and usnic acid) (6). Usnic acid was shown to inhibit the growth of a wide range of mycobacterial species (*Mycolicibacterium aurum*, *Mycolicibacterium avium*, *Mycolicibacterium chelonae*, *Mycolicibacterium fortuitum*, *Mycolicibacterium kansasii*, and *M. tuberculosis* H37Rv) (7–11). Its general antibacterial activity was related to the increase in bacterial membrane permeability (12), inhibition of the efflux pumps and nucleic acid synthesis (13), or alterations in fatty acids and peptidoglycan production (14). Our previous study revealed that the antimycobacterial activity of usnic acid was manifested by disturbances in the redox homeostasis and by increased production of structural elements of the cell wall and cell membrane in *M*. *tuberculosis* H37Ra (15).

In recent years, transcriptomic analysis has gained much interest in drug discovery and development. Its usefulness in determining the molecular mechanism of a drug’s action results from the possibility of monitoring specific gene expression patterns induced by the drug (16). Synthetic chemicals or natural products can bind to a particular drug target but also can nonspecifically affect cellular signal transduction, resulting in changes in metabolic pathways (17). Moreover, natural products can alter motility, biofilm formation, and the expression of virulence genes (18). Sequencing technologies realize the untargeted monitoring of the cellular transcriptomic signatures, which provide high-dimensional omics data. This massive data captures the complexity of biological systems, reveals the mode of action of drug/potential drug, and offers the possibility of predicting susceptibility to known therapeutics (16). RNAseq enables the exploration of drug-induced genome-wide gene expression shifts, with even greater precision achieved through single-cell RNAseq, providing valuable information for drug and biomarker discovery (19). Metabolomics, as a sister technique of transcriptomics, also plays a crucial role in capturing changes in cellular metabolism. It does so by revealing the changes in the production or utilization of low-molecular-weight compounds, which can be starting, intermediate, or end products of metabolic transformations (20). The practical application of metabolomics enables the determination of the modes of action of natural products and the mechanisms of cell adaptation and resistance in response to stress agents (21, 22). In the case of mycobacteria, metabolites of significant importance are lipids. Mycobacteria produce several classes of lipids that play structural and functional roles. Constituting 40% of the dry cellular weight, lipids contribute to cell envelope formation, energy storage, virulence, pathogenicity, and persistence (23). Therefore, lipidomics gives insight into crucial aspects of the mycobacterial compartment of the cellular metabolic activity. Numerous studies have demonstrated the power of the transcriptomic-metabolomic combined approach in determining the antibacterial mechanism of action of known antimicrobials and new molecules at both metabolomic and transcriptomic levels (24, 25). This approach has also been used to study compounds with different activities for potential drug repurposing as antimicrobials, indicating that one drug can target more than one molecular target (26).

The combined transcriptomic-metabolomic approach was applied in this work to elucidate the strain-specific metabolic response of virulent *M. tuberculosis* H37Rv and to compare it with the previously determined influence of usnic acid on the avirulent H37Ra strain. Since the MIC value of usnic acid against H37Rv was different than against H37Ra (8 µg/mL and 32 µg/mL, respectively), it indicated unlike susceptibility of the sister strains against the same stressing factor. Considering the genetic and phenotypic differences between both strains, we aimed to explore the possible metabolic dissimilarities occurring under the influence of usnic acid. H37Ra responded with increased metabolic activity, whereas H37Rv reduced basic metabolic processes and activated alternative iron-dependent energy production. These differences reflect and underscore the diverse susceptibility of sister strains of the same species to the same antibacterial agent and highlight iron metabolism as the important drug target in mycobacteria.

## RESULTS

### Usnic acid-induced metabolic changes—the altered lipid profile of *M. tuberculosis*

**Fig 1:**
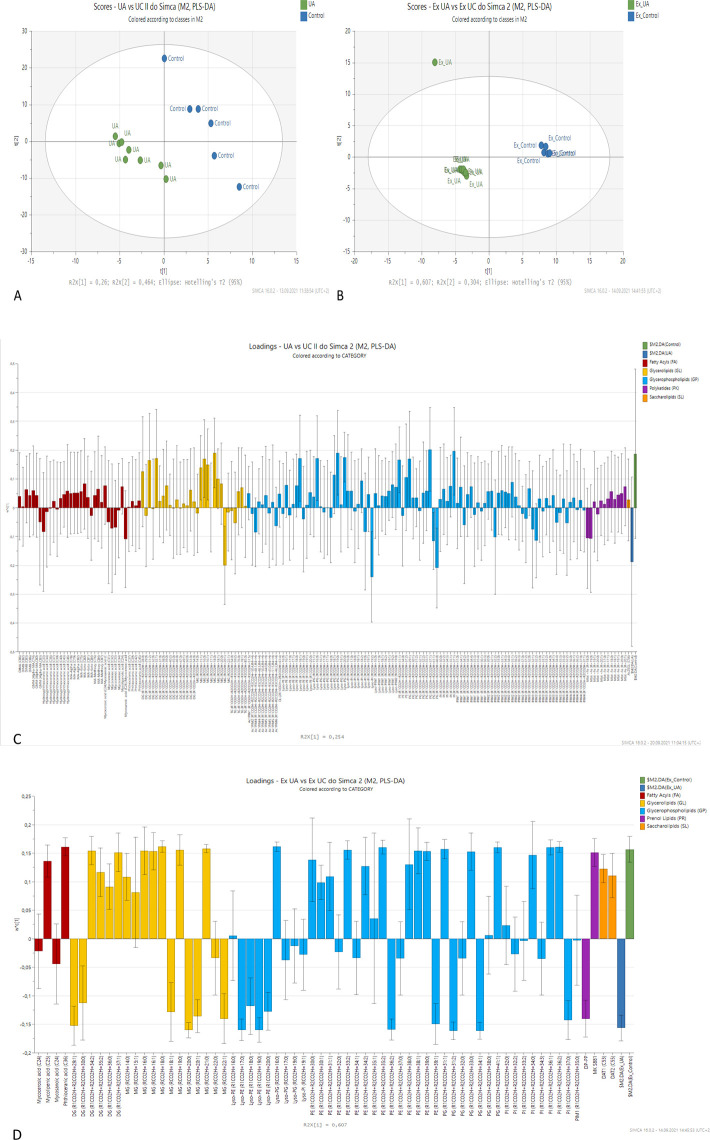
The score plot of PLS-DA of lipidomic profiles recorded for whole-cell extracts (A) and extracellular fraction (B) shows the separation between classes. The ellipse represents the Hotelling T 2 with a 95% CI; R2X explains variation in X by the component. PLS-DA loading plots of whole-cell extracts (C) show that the usnic acid-treated group has lower levels of lipids, while in the extracellular fraction (D) downregulation was less pronounced; control (+) vs treated sample (−) values; Ex, extracellular; UA, usnic acid-treated bacteria.

**Fig 2:**
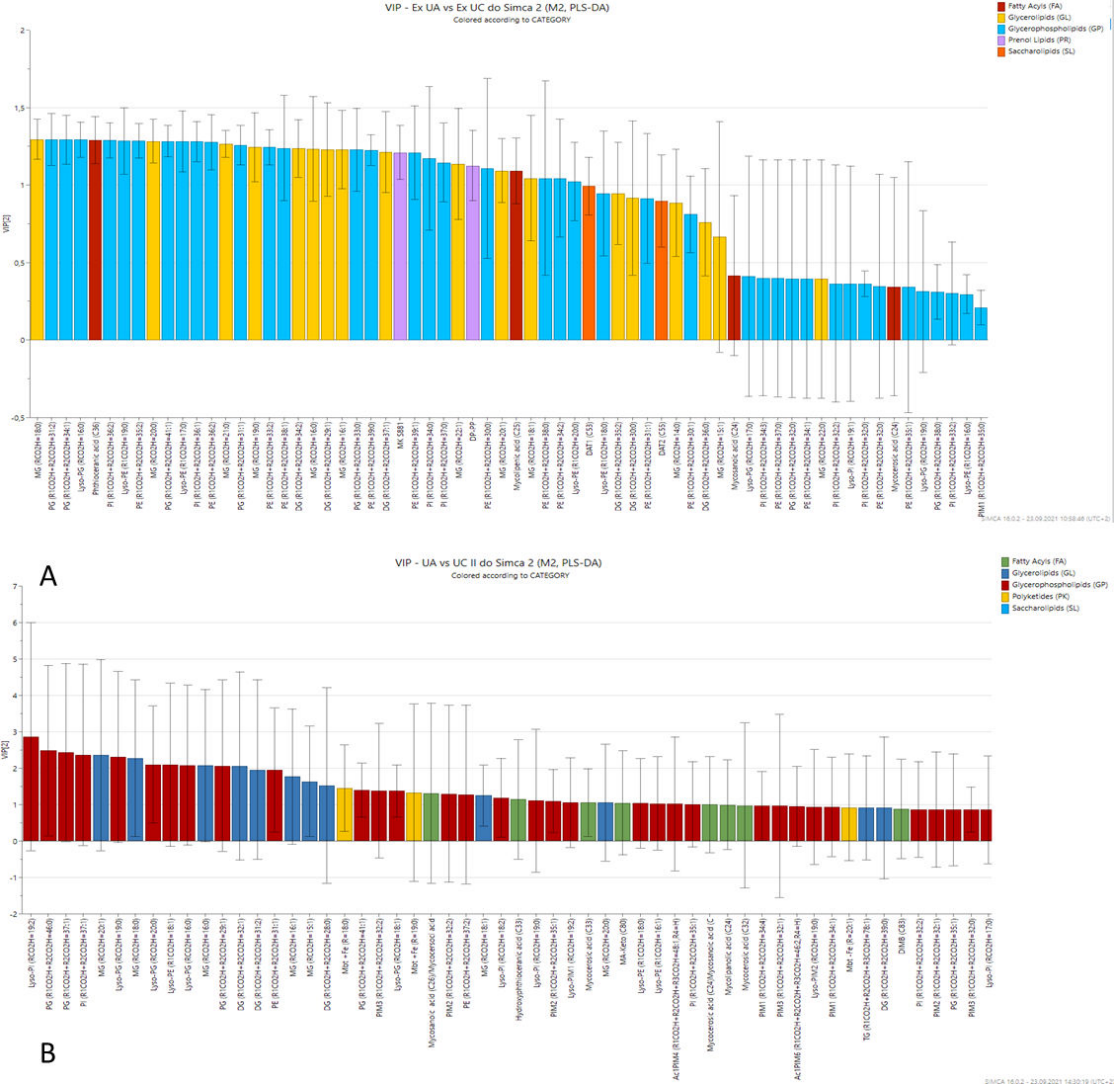
VIP plot of extracellular (A) fraction and whole-cell fraction (B). The most influential variables, with scores greater than 1, were considered significant: mainly glycerophospholipids with lipid chains having more than 30 carbons in the extracellular fraction, glycerophospholipids, glycerolipids, their lyso form, and mycobactins in cellular extracts.

The applied partial least squares-discriminant analysis (PLS-DA) enabled us to obtain the data dimensionality reduction with the assignment of the class labels. As shown in Fig. 1A and B, both groups of samples (whole-cell extracts and extracellular fraction) were clearly separated from the controls by principal component 1. The additional separation by principal component 2 was achieved for extracellular fraction samples, although the number of lipids identified in these samples was much smaller than in whole-cell extracts. The two first components explained 95% of the total variance in the data. Variable importance in projection (VIP) scores, which indicate the contribution of each variable in the PLS-DA model, showed that compounds from classes of glycerolipids, glycerophospholipids, and prenol lipids contributed the most to the differences between the extracellular fraction of the test and control group (Fig. 2A). In the whole-cell extracts, glycerophospholipids and glycerolipids, as well as polyketides and fatty acyls (FAs), had VIP scores between 1 and 3 (Fig. 2B), indicating that the content of these compounds was significantly different in both groups of samples.

The relevant loading column plots showed that usnic acid treatment resulted in significant metabolite changes compared to the control group (Fig. 1C and D). The majority of lipids detected in the whole-cell fraction were downregulated. Only a few individual compounds were present in higher amounts in test samples. In FAs class phthiocerol dimycocerosates (DIMA) and phthiodiolone dimycocerosates (DIMB), mycolic acids and phthioceranic acids were downregulated in UA-treated samples. Some upregulation for mycocerosic/mycosanoic acids was noticed. Glycerolipids were depleted in test samples with significant monoacylglycerols (MGs) and diacylglycerols (DG) downregulation. In the largest class of glycerophospholipids, only a few molecules were significantly changed in the subclasses of monoacylglycerolphosphoethanolamines (Lyso-PE), monoacylglycerophosphoglycerols (Lyso-PG), and monoacylglycerophosphoinositols, while the majority of lipids were only slightly affected. Interestingly, in the polyketides class, only mycobactins were assigned, among which three Mbt + Fe (containing iron) were significantly upregulated, whereas all Mbt – Fe (empty mycobactins) were decreased. The diacylated sulfolipid (Ac2SGL, C56) was downregulated in usnic acid-treated samples (Fig. 1C).

In the extracellular fraction (Fig. 1D), two of the four detected fatty acids were decreased in test samples. Also, most glycerolipids (MG and DG) were observed in lower amounts in test samples. In the class of glycerophospholipids, slight fluctuation was noticed. Lyso-PE was upregulated, while one Lyso-PG was downregulated. Also downregulated were diacylglycerolphosphoethanolamines (PE) and most diacylglycerophosphoinositols. Bactoprenol diphosphate was enriched under usnic acid treatment, but ubiquinone MK S881 and 2,3-di-O-acyltrehaloses DAT1 (C53) and DAT2 (C55) were depleted in stressed bacteria extracellular fraction.

### Transcriptomic changes induced by usnic acid

**Fig 3:**
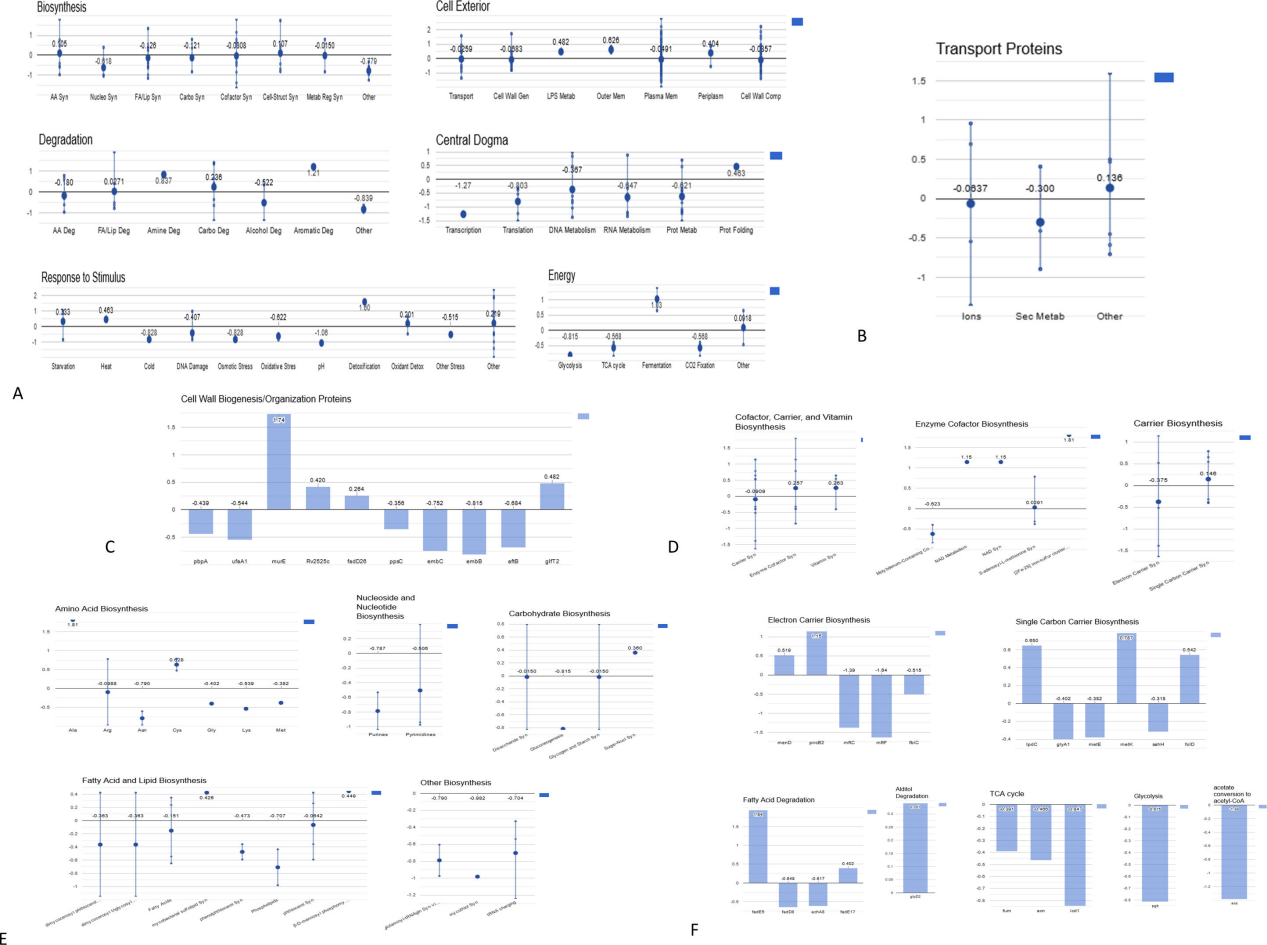
The expression profiles of genes coding proteins involved in various cellular processes in *M. tuberculosis* H37Rv under the influence of usnic acid. After 24 h of exposure, the genetic material was extracted and subjected to RNAseq. The expression was presented as a fold change of the test group (usnic acid treated, 512 µg/mL) in relation to the control group (untreated). The visualization was done using the Omics Dashboard (BioCyc). The panels represent top-level cellular systems, each containing a set of plots that represent component subsystems. These subsystems map to one or more pathways or pathway classes, Gene Ontology (GO) terms, or computed categories based on transport or regulatory activity. Global transcriptomic response (A); transport proteins (B); cell wall biogenesis/organization proteins (C); enzyme cofactors, carriers (D); biosynthesis processes (E); energy generation (F).

The RNAseq analysis revealed the global transcriptomic response of bacteria under the influence of usnic acid. The expression of genes coding proteins related to main cellular processes was differently altered (Fig. 3A through F). Biosynthetic pathways showed downregulation, which was also more pronounced in the case of energy, transcription, and translation. The expression of genes coding proteins involved in degradation processes and response to stimuli was changed differently. The expression of genes coding proteins working in the cell exterior was generally slightly upregulated but varied depending on process/pathway type. The used dose of usnic acid caused some inhibition in metabolic processes. However, several exceptions were observed among the majority of downregulated biosynthesis pathways. Genes responsible for alanine and cysteine biosynthesis, cofactor, carrier, vitamin biosynthesis, and peptidoglycan biosynthesis were upregulated, suggesting that bacteria activated some metabolic response to the stressing agent.

### Usnic acid changes cell wall biogenesis/organization

In the group of genes related to cell wall biogenesis/organization proteins, the highest fold change was observed for *mur*E (1.74 up) involved in the biosynthesis of bacterial cell wall peptidoglycan (27). The upregulation of other genes (*pks*12, *fad*D26, *fad*D28, *pks*13, and *glf*T2) did not exceed 0.5-fold. Downregulation of 1.15-fold was described for *mas*, a gene coding multifunctional mycocerosic acid synthase membrane-associated, needed in the almost last step in dimycocerosyl phthiocerol and dimycocerosyl phenolphthiocerol biosynthesis (27). Downregulation higher than 0.5-fold was also observed for *ufa*A1, *pps*A, *emb*C, *emb*B, and *aft*B (Fig. 3C).

In the case of peptidoglycan formation, besides *mur*E coding UDP-*N*-acetylmuramoyl-L-alanyl-D-glutamate–2,6-diaminopimelate ligase, which catalyses the addition of meso-diaminopimelic acid to the nucleotide precursor UDP-N-acetylmuramoyl-L-alanyl-D-glutamate (27) forming UDP-MurNAc-L-Ala-γ-D-Glu-*meso*-A_2_pm = UDP-*N*-acetyl-α-D-muramoyl-L-alanyl-γ-D-glutamyl-*meso*-2,6-diaminopimelate, also *csd* contributed to the possible strengthening of this cell wall layer in bacteria exposed to usnic acid. *csd*, the expression of which increased 1.81-fold, controls cysteine desulfurase, yielding alanine, an essential element of peptidoglycan structure. Alanine is present in short peptides (l-alanyl-γ-d-isoglutamyl-meso-diaminopimelate-d-alanyl-d-alanine) connecting adjacent glycan strands (28).

Exposure to usnic acid altered the expression of genes, steering the formation of arabinogalactan, a biopolymer consisting of arabinose and galactose residues. The slight upregulation (0.36-fold) of sugar nucleotide synthesis was represented by *cps*Y gene coding UDP-glucose-4-epimerase, the enzyme needed to produce UDP-α-D-galactose for galactan. Also, 0.48-fold upregulation was observed for *glf*T2 coding arabinogalactan UDP-galactofuranosyltransferase 2, which enables the formation of the full galactofuran chain, by the attachment of new galactofuranosyl units to the terminal galactosyl residue of the nascent galactan chain (29). A different situation was noticed for genes involved in forming the arabinan compartment of arabinogalactan and lipoarabinomannan. *emb*B, *emb*C, and *aft*B were downregulated by 0.75-, 0.81-, and 0.68-fold, respectively. *emb*B is involved in arabinofuran transfer to arabinan. At the same time, the function of *emb*C is related to arabinofuran transfer to lipoarabinomannan (30). *aft*B [coding β(1→2) arabinofuranosyltransferase] catalyses the attachment of terminal β(1→2) arabinofuranosyl residues to the arabinan chains of arabinogalactan and lipoarabinomannan, marking the end point of arabinan synthesis before the molecule is decorated with mycolic acids or caps, respectively (27).

Also, the expression of the *ufa*A1 gene, which controls cyclopropane-fatty-acyl-phospholipid synthase, creating cyclopropane rings in fatty acyl chains, was lowered by 0.54-fold. In mycobacteria, mycolic acids, which are attached to arabinogalactan, often contain cyclopropane groups. The role of these rings is to stabilize the membranes by increasing the ordering of the chains without sacrificing membrane fluidity (27). Cyclopropane rings can also be created in the phospholipid bilayer (cell membrane) to decrease the proton permeability of phospholipid bilayers as a response to stress conditions (27). The downregulation of *ufa*A1 was accompanied by the alteration in the expression of *fad*D26, *fad*D28, *pps*A, *pps*C, and *mas* genes controlling the synthesis of dimycocerosyl phthiocerol biosynthesis and dimycocerosyl triglycosyl phenolphthiocerol biosynthesis. Fatty-acid-AMP ligases (coded by *fad*D26 and *fad*D28) and type-I polyketide synthases (coded by *pps*A and *pps*C) are needed for phthiodiolenone synthesis, an intermediate in phthiocerol biosynthesis (27). The expression of *fad*D26, producing (behenoyl)adenylate, and *fad*D28, creating behenoyl-[(phenol)carboxyphthiodiolenone synthase] complex was slightly increased (by 0.26- and 0.42-fold, respectively) under usnic acid treatment. However, the downregulation was observed for the later step in phthiodiolenone biosynthesis, the creation of C34-carboxyphthiodiolenone-[(phenol)carboxyphthiodiolenone synthase] by *pps*A and *pps*C (by 0.35- and 0.59-fold, respectively). Phthiodiolenone is an intermediate in phthiocerol biosynthesis, which combined with mycocerosyl-(mycocerosic acid synthase) complex produced by mas, finally yields a dimycocerosyl phthiocerol by the action of *pap*A5 (27). The expression of mas was lowered in this experiment, which resulted in the observed decreased production of DIMA and DIMB. However, as revealed by metabolomic analysis, the content of mycocerosic acids was increased in extracts from bacteria exposed to usnic acid. This may suggest that the produced mycocerosic acids were not incorporated into phthiocerol molecules.

Taking into account the observations described above, the upregulation of genes involved in the formation of cell wall structures close to the cell interior and the downregulation of those responsible for distal compartments was noticed.

### Usnic acid affects transporters

The influence of usnic acid was also visible in the changed expression of genes coding transmembrane transporters (Fig. 3B). Downregulation was noticed for *mmpL*11, *mmpL*2, *mmpL*1, *mmpL*5, *atp*A, and *ctp*D (by 0.73-, 0.35-, 0.78-, 0.41-, 0.54-, and 0.45-fold, respectively), while the expression of *mmpL*5, *lpq*Y, and *amt* increased (by 0.40-, 0.92-, and 0.95-fold, respectively). The function of MmpL1 and MmpL2 transport proteins is not defined yet, whereas MmpL11 transports long-chain triacylglycerols (TGs) and mycolate wax esters outside the plasma membrane (31). Downregulated *ctp*D codes Fe2+-exporting P-type ATPase, which excretes iron ions from the cell (27), while upregulated *lpq*Y (trehalose-binding lipoprotein) and amt (putative ammonium transporter) control transfer of trehalose and ammonium to the cell interior (27). MmpL4 and MmpL5 (working together with MmpS5 and MmpS5) are two different siderophore export systems, whose importance is dependent on the environment/infection context. MmpL5 also works as a drug efflux system (31). The upregulation of mmpL4 and downregulation of *ctp*D may indicate the increased demand for iron ions in bacterial cells. In contrast, the downregulation of *mmpL*11 and *mmpL*5 suggests a decrease in transmembrane transport of cell envelope elements and xenobiotics in bacteria exposed to usnic acid. Moreover, the overexpression of genes involved in the salvage of trehalose and the assimilation of ammonium may be related to the redirection of energy production caused by usnic acid.

### Influence on enzyme cofactors, energy generation, and carrier biosynthesis

Among genes related to the enzyme cofactor biosynthesis (Fig. 3D and F), the highest upregulation caused by usnic acid was observed for *csd* (1.81-fold, coding cysteine desulfurase) involved in [2Fe-2S] iron-sulfur cluster biosynthesis and in L-alanine biosynthesis III and for *Rv1462* gene coding Rv1462 protein involved in iron-sulfur cluster assembly. Iron-sulfur (Fe-S) clusters are cofactors of iron-sulfur proteins (e.g., ferredoxins), consisting of a cluster of two to four iron atoms linked to sulfides, some of which are cysteine (cysteine biosynthesis gene was also upregulated) residues in the protein, which function as intracellular electron carriers and also participate in the activation of substrates, the stabilization of radicals and structures, the protection of proteins from enzymes, and the storage of iron and sulfur (27, 32). Additionally, overexpression was noticed for *fpr*B gene coding ferredoxin/ferredoxin—NADP reductase (iron-sulfur cluster binding) underlying the increased role of mechanisms involving iron in bacteria under usnic acid exposure.

The other highly upregulated gene (1.14-fold; Fig. 3F) was *pnc*B2 coding nicotinic acid phosphoribosyltransferase. This enzyme is involved in NAD salvage pathway I, restoring the cell’s NAD pool. NAD and its phosphorylated derivative, NADP, are two of the most essential coenzymes in redox reactions in the cell. Generally, NAD is involved in catabolic reactions, while NADP in anabolism (27).

The catabolic process was confirmed by the upregulation of genes controlling lipid degradation. The *glp*D2 gene coding glycerol-3-phosphate dehydrogenase two involved in glycerol (alditol) and glycerophosphodiesters degradation pathways was overexpressed by 0.34-fold. Also, the expression of *fad*E5 and *fad*E17 genes involved in the fatty acids β-oxidation by acyl-CoA dehydrogenases was increased by 1.84- and 0.40-fold, respectively (Fig. 3F). Acyl-CoA dehydrogenases degrade long-chain acyl-CoA molecules to acetyl-CoA and propionyl-CoA. The metabolomic study showed decreased fatty acyl levels, which was also consistent with lowered expression of *fad*D8 (0.64-fold down; Fig. 3F) coding fatty-acid-CoA synthetase.

On the other hand, the *Rv1760* gene coding diacylglycerol O-acyltransferase was upregulated by 0.65-fold, while *lip*F steering enzyme of pospholipase C activity (putative triglyceride lipase activity) was downregulated by 1.05-fold. Diacylglycerol O-acyltransferase transfers the third acyl unit to 1,2-diacyl-sn-glycerol creating a TG molecule. The overexpression of *Rv1760* putatively suggests an increase in the formation of TG, while downregulation of *lip*F degrading TG may indicate their decreased catabolism. Nevertheless, this observation is inconsistent with the above increase in fatty acids β-oxidation. Also, metabolomic analysis does not provide a clear view because all subgroups of glycerolipids were downregulated.

Acetyl-CoA molecules generated during fatty acid β-oxidation can be further utilized in the tricarboxylic acid (TCA) cycle. However, in this experiment, genes coding for enzymes participating in the TCA cycle were downregulated under the exposure of usnic acid. The expression of *fum*, *acn*, and *icd1* was lowered by 0.39-, 0.46-, and 0.84-fold, respectively (Fig. 3F). This indicates the reduction of biochemical reactions performed not only within the TCA cycle but also in the glyoxylate shunt, as fumarase and aconitase participate in both cycles (27). Moreover, besides *icd1*, two other gene-coding NADH dehydrogenases: *Rv1812c* and *nuo*N, and *atp*A gene controlling ATP synthase F1 complex subunit, involved in pumping H^+^ ions and generating proton motive force for ATP production (27, 33) were downregulated by 0.60-, 0.70-, and 0.54-fold, respectively, indicating that the primary electron transfer mechanism was repressed in bacteria under usnic acid treatment.

Additionally, the *acs* gene controlling the reaction of acetate conversion to acetyl-CoA by acetyl-coenzyme A synthetase was downregulated by 1.38-fold, and two genes controlling glycolysis were also downregulated (*pgk* by 0.81-fold and *pyk* by 0.58-fold), indicating that fatty acids β-oxidation was favored as a source of acetyl-CoA in bacteria subjected to usnic acid treatment.

Moreover, a 1.01-fold upregulation was noticed for the *pck*G (*pck*A) gene controlling phosphoenolpyruvate carboxykinase involved in gluconeogenesis and cellular response to iron ion starvation (27) and for the *cyp*132 gene (by 1.2-fold) coding putative cytochrome P450 132 protein with iron ion binding and oxidoreductase activity underlining again the role of iron in bacteria response to usnic acid.

The genetic regulation of carrier biosynthesis was altered by usnic acid. The expression of genes coding for electron carrier formation was more dysregulated than genes involved in the biosynthesis of single-carbon carriers (Fig. 3F). The highest upregulation (1.14-fold) was noticed for *pnc*B2 controlling nicotinic acid phosphoribosyltransferase. Upregulated (0.51-fold) was also the *men*D gene coding for bifunctional menaquinone biosynthesis protein, acting at the early step of menaquinone biosynthesis. Interestingly, the metabolomic analysis revealed a lowered level of MK-S881 (sulfated dihydromenaquinone-9), which was proposed as an intermediate in a mechanism of non-transcriptional regulation of quinone availability (34). Another observation was a significant downregulation of *mft*C and *mft*E genes (by 1.38- and 1.64-fold, respectively) controlling the synthesis of mycofactocins, a family of related electron acceptors (27), and a 0.51-fold decrease in the expression of the *fbi*C gene, needed at the early step of factor 420 (redox-active compound) biosynthesis.

In the case of single-carbon carrier S-adenosyl-L-methionine (SAM) biosynthesis, the observed genetic alterations showed some downregulation of *sah*H (coding adenosylhomocysteinase, by 0.31-fold; Fig. 3F) and *met*E (5-methyltetrahydropteroyltriglutamate–homocysteine methyltransferase, by 0.38-fold; Fig. 3F), which control the first two reactions in the S-adenosyl-L-methionine cycle II and lead to the formation of L-methionine. However, the gene *met*K, which supervises the later step (the conversion of L-methionine to S-adenosyl-L-methionine), was upregulated by 0.78-fold. The genetic control of biosynthesis of the other one-carbon unit carrier, the folates (vitamin B9), was also upregulated. The expression of *lpd*C (coding dihydrolipoamide dehydrogenase subunit) and *fol*D (coding bifunctional protein FolD: methylenetetrahydrofolate dehydrogenase + methenyltetrahydrofolate cyclohydrolase) involved in N10-formyl-tetrahydrofolate biosynthesis increased by 0.65- and 0.64-fold, respectively. The upregulation of genes controlling SAM and folate biosynthesis suggests the increased demand for molecules acting as carriers of one-carbon units in various oxidation states (27).

## DISCUSSION

The characteristic feature of mycobacteria is its specific cell envelope composition. Three distinct layers (cell membrane, mycomembrane, and capsulae) are formed predominately by lipids and their conjugates with sugars (35). Metabolomic insight into the state of the cell envelope gives the possibility to assess if the used stressing agent acts at this level. The altered lipid profile of bacterial cells and extracellular fraction observed under the influence of usnic acid (downregulation of glycerolipids, glycerophospholipids, and prenol lipids) suggests the general decrease of cellular metabolic activity and excludes the leakage/release of lipids into the culture medium. At the same time, the increased mycobactins containing iron indicate the importance of iron in the generated stress conditions.

Since metabolic observations were limited to lipids, the untargeted transcriptomic analysis was performed to get global insight into the state of the mycobacterial cells subjected to usnic acid treatment. The described metabolomic findings correlated with dysregulations in gene transcription. Exemplarily, the lowered levels of DIMA and DIMB (molecules contributing to cell wall impermeability [36–38]) were accompanied by the downregulation of *mas*, a gene encoding the multifunctional mycocerosic acid synthase, membrane-associated enzyme needed in the almost last step of dimycocerosyl phthiocerol and dimycocerosyl phenolphthiocerol biosynthesis (27). Additionally, the transcriptomic analysis showed that fatty acid β-oxidation was favored as a source of acetyl-CoA in bacteria subjected to usnic acid treatment. This can also be a reason for a diminished synthesis of lipid molecules containing fatty acids.

Importantly, the main electron transfer mechanism within the TCA cycle and glyoxylate shunt was repressed (*fum*, *acn*, *icd1*, *Rv1812c*, *nuo*N, and *atp*A were downregulated) in bacteria under usnic acid treatment. The overexpressed *cyp*132 and *fpr*B indicated that bacteria activated alternative electron transport ways: cytochrome P450 and FprB systems. Increased expression of ferredoxin reductase FprB gene correlated with (i) overexpression of *csd* involved in the synthesis of iron-sulfur (Fe-S) clusters, a cofactor of iron-sulfur proteins, which are intracellular electron carriers; (ii) downregulation of *ctp*D coding Fe^2+^-exporting P-type ATPase, which excretes iron ions from the cell; and (iii) increased levels of mycobactins containing iron. Under the influence of usnic acid, the pool of iron bound to mycobactins was used for iron-sulfur cluster biosynthesis, and their binding to ferredoxin/ferredoxin—NADP reductase to be used in cytochrome P450 for energy generation. Such an alternative way for energy production was already described in *M. tuberculosis* entering into a dormant-like phenotype in the lack of iron when the blockage found for NADH dehydrogenase could be replaced by the cytochrome P450 and FprB system in the generation of energy sources (39). Although, in our experiment, bacteria were not iron deprived, usnic acid caused a shift from oxidative phosphorylation to energy production in cytochrome P450. The protective role of the bd unit of cytochrome P450 against antibiotic treatment was already described. Cytochrome bd was induced in *M. tuberculosis* in response to nitric oxide, potassium cyanide, and uncouplers, as well as various anti-infectives such as triclosan, antifungal azoles, or chlorpromazine (40). Moreover, the induction of cytochrome bd-type menaquinol oxidase (subunit I) gene was observed in *M. tuberculosis* and *Mycobacterium marinum* under the influence of various classes of antibiotics, in particular inhibitors of oxidative phosphorylation (41, 42) and cell wall inhibitors (41). Hence, the putative target of usnic acid in mycobacterial cells may involve interference with iron metabolism, inhibition of oxidative phosphorylation, cell wall synthesis inhibition, or all of them.

Interestingly, metabolic and transcriptomic changes observed in the virulent *M. tuberculosis* H37Rv strain significantly differed from previously described alterations caused by usnic acid in the avirulent H37Ra strain. Under the influence of the same dose of usnic acid (512 µg/mL), H37Ra responded to in increase in molecules related to the restoration of redox balance (factor 420) and the rearrangements of cell envelope (higher levels of lipids) (15), whereas H37Rv diminished synthesis of lipid molecules and redirected energy production. So, significant differences in bacteria response may be attributed to distinct susceptibility to the stressing agent. The same effective dose caused dynamic rearrangement of bacterial metabolism in less susceptible strains to overcome the stressing agent’s influence. In contrast, the more sensitive strain was, in a certain way, metabolically suppressed. The susceptibility to antimycobacterial compounds is undoubtedly related to existing differences between H37Rv and H37Ra genomes, including 53 insertions and 21 deletions in H37Ra relative to H37Rv. Mutations affecting transcription factors and/or global metabolic regulations in H37Ra result in higher *in vitro* survival under aging stress (43). These mutations may also contribute to lower susceptibility to xenobiotics *in vitro*. The additional mutations affecting cell envelope, primary metabolism, *in vivo* growth, and variations in the PE/PPE/PE-PGRS family genes are thought to be a basis of virulence attenuation in H37Ra (43), but at the same time, they may be a reason for a distinct metabolic response to stressing factors, such as usnic acid. The expression of genes related to energy metabolism, cofactor biosynthesis, nucleotide metabolism, lipid metabolism, protein degradation, and sigma factors (*sigC*, *nrdH*, *phoH2*, *pabB*, and *lpdA*) in H37Ra vs H37Rv was increased *in vitro* but decreased in macrophages (43). This information is in agreement with the lower sensitivity of H37Ra to usnic acid *in vitro* in comparison to the sensitivity of H37Rv.

## MATERIALS AND METHODS

### Tested organism, culture conditions, MIC, and effective dose determination

Reference virulent *M. tuberculosis* H37Rv, ATCC 25618 strain, was used in the experiments. Investigations were performed in 96-well microtiter plates by the twofold serial microdilution of the tested compound using Middlebrook 7H9 Broth medium (Beckton Dickinson) containing 10% of Middlebrook oleic albumin dextrose catalase (OADC) enrichment growth supplement (Beckton Dickinson). The bacterial mass was transferred to 5 mL of the 7H9 medium and vortexed with glass beads for 3 min. After 30 min of sedimentation at room temperature, the upper phase was transferred to a sterile tube and left for 15 min. One milliliter of supernatant was placed in a sterile tube, adjusted to 0.5 McFarland standard with OADC-supplemented Middlebrook 7H9 broth, and diluted to 1:100. The final amount of bacteria was 1.5 × 10^7^. The stock solution of usnic acid (98% purity, Merck, Darmstadt, Germany) was prepared in dimethyl sulfoxide (DMSO, Millipore Sigma, St. Louis, MO, USA) at 1,024 µg/mL concentration. The solution was diluted serially in sterile 96-well microtiter plates using 100 µL Middlebrook 7H9 Broth medium with OADC. Subsequently, 100 µL of microbial suspension was added to each well. The final inoculum of *M. tuberculosis* was approximately 5 × 10^5^ CFU/mL in concentrations of usnic acid that ranged from 0.25 to 512 µg/mL. The final of DMSO concentration did not exceed 2% (vol/vol). A growth control containing no antibiotic and a sterile control without inoculation were also prepared on each plate. The plates were incubated at 37°C for 3 weeks. After incubation, 30 µL of Alamar Blue solution was added to each well, and the plate was re-incubated for 24 h (44). The growth was indicated by the color change from blue to pink. The lowest concentration of a compound that prevented the color change was considered its MIC. Isoniazid (INH), rifampicin (RMP), streptomycin (SM), and ethambutol (EMB; MilliporeSigma, St. Louis, MO, USA) were used as reference drugs. The MIC value of usnic acid was 8 µg/mL, while the MIC of INH, RMP, SM, and EMB were 0.125 µg/mL, 0.0625 µg/mL, 0.25 µg/mL, and 0.5 µg/mL, respectively.

### Bacterial exposure to usnic acid

For the metabolomic experiment, metabolically active bacteria in the late logarithmic growth phase (approx. 10^9^ CFU/mL) were used. The effective dose of usnic acid in high-density culture was determined with Alamar Blue solution. It was higher than the MIC value determined for a much less dense culture (5 × 10^5^ CFU/mL), and it equaled 512 µg/mL. Two flasks with 400 mL of Middlebrook 7H9 liquid medium supplemented with OADC enrichment were started with 4 mL of freshly prepared inoculum. Bacteria were grown at 37°C with aeration of 100 rpm. Because *M. tuberculosis* is a slow-growing organism, which divides every 20–24 hours (45), 4–5 incubation weeks were needed to obtain the cell density around 1 × 10^9^ CFU/mL (average 800 mg of dry biomass per flask). The test culture (400 mL) was supplemented with usnic acid to a concentration of 512 µg/mL (normalized by weight, dissolved in DMSO, DMSO did not exceed 2% in the culture), while DMSO control (another 400 mL culture) was grown with 2% DMSO. The cultures were incubated for 24 hours. Then, bacterial metabolism was stopped, and metabolites were quenched by the addition of cold methanol (−60°C) in the volume equal to the volume of the culture (1:1 [vol/vol]). Next, the cultures were aliquoted in 50 mL Falcon tubes and centrifuged for 30 min at 8,000 rpm at 4°C. The supernatant was collected, while bacterial pellets were rinsed three times with cold phosphate-buffered saline pH 7.4 (Biomed, Lublin, Poland) and centrifuged again to remove traces of the medium. The bacterial biomass was frozen in liquid nitrogen (−80°C), lyophilized (SRK-Systemtechik GmbH, Germany), weighed, and stored at −20°C before extraction and analysis. The supernatant was also stored at −20°C until extracted. All bacterial manipulations were made in a BSL3 laboratory.

### Total RNA extraction

The *M. tuberculosis* cells were collected by centrifugation of 2 mL of a culture that had been exposed to usnic acid. The exact number of cells was harvested from the DMSO-incubated culture. The bacterial pellet was then resuspended with 1 mL of RNA pro solution (MP Biomedicals, Santa Ana, CA) and placed in a tube containing 0.8 mL zirconia beads (0.1 mm diameter). The cells were disrupted in a bead-beater (FastPrep24 instrument; MP Biomedicals, Santa Ana, CA) at the highest speed using two 45-second pulses. Afterward, the cell remains were spun down, and the liquid was transferred to a new tube. Total RNA isolation was performed with the FastRNA Pro Blue kit (MP Biomedicals, Santa Ana, CA) according to the manufacturer’s manual. DNase I (MilliporeSigma, St. Louis, MO, USA) was used to eliminate possible genomic DNA contamination of RNA preparations. The RNA concentration and purity were determined by spectrophotometric measurement using the Biotek Synergy H1 microplate reader (BioTek Instruments, USA), ensuring an A_260_/A_280_ ratio between 1.8 and 2.1. The samples were aliquoted and stored at −80°C for future use.

### RNAseq

Sequencing reactions were performed by Genomed S.A. (Warsaw, Poland) using the BigDye Terminator v3.1 kit from Applied Biosystems (Life Technologies). Sequencing reaction products were separated on a 3730xl DNA Analyzer capillary sequencer. The study material consisted of RNA isolated from six samples, divided into two groups (control/usnic_acid), originating from *M. tuberculosis* H37Rv. The analyzed samples had three biological replicates. The sequencing results included a transcriptome sequence read in the FASTQ file format (paired reads, Table 1).

**TABLE 1:** Description of the samples included in the experiment and the number of pairs of readings obtained for each sample

Sample number	Sample ID	Group	Replicate	Number of read pairs
1	Control 1	Control	1	16,730,858
2	Control 2	Control	2	18,051,461
3	Control 3	Control	3	13,488,706
4	Usnic acid 1	Usnic_acid	1	17,542,557
5	Usnic acid 2	Usnic_acid	2	16,989,326
6	Usnic acid 3	Usnic_acid	3	17,378,845

The bioinformatic analysis aimed to assemble transcripts, calculate gene expression profiles for individual samples, and compare gene expression profiles between control and experimental groups, including selecting genes with statistically significant differences in expression. Transcript annotations were also performed based on homology to known protein sequences from the Uniprot database (https://blast.ncbi.nlm.nih.gov/Blast.cgi). Gene ontology identifiers were also determined, which were overrepresented in the set of differential genes for each analyzed comparison. The global gene expression data were visualized by the Omics Dashboard for interactive exploration of gene-expression data (46).

### Lipid extraction

Extracellular lipids were extracted from the culture medium with cold methanol. After removing the bacteria by centrifugation, the supernatant (500 mL) was concentrated to 200 mL volume and extracted with three portions of a mixture of chloroform and methanol (2:1 [vol/vol]; 3 × 150 mL). The pooled organic phase was evaporated to dryness. The fatty residue was then solubilized in acetonitrile-methanol-isopropanol (1:1:2 [vol/vol/vol]) at 1 mg/mL concentration and analyzed (10 replicates for test and 5 for control samples). The culture medium without bacteria was extracted similarly as a control background. After the evaporation of the organic phase, no liquid fatty residue was obtained in this sample.

Whole-cell lipids were extracted from bacteria-lyophilized biomass with a mixture of chloroform-methanol-water (1:1:0.1 [vol/vol/vol]). Each sample (ca. 30 mg of dry biomass; nine test samples and six controls) was sonicated with 1.5 mL of extracting mixture for 20 min without heating. After centrifugation (15 min at 13,000 rpm at 4°C), clear supernatant was collected, and the extraction procedure was repeated. The supernatants were transferred to clean Eppendorf tubes and evaporated under reduced pressure at 30°C. The obtained dry residue was dissolved in methanol-acetonitrile-isopropanol (1/1/2 [vol/vol/vol]), filtered through polytetrafluoroethylene membrane syringe filters, and subjected to liquid chromatography-mass spectrometry analysis.

### Lipids analysis

Lipids were chromatographically separated on Agilent 1200 Infinity HPLC (Agilent Technologies, Santa Clara, CA, USA) equipped with Gemini column (3 µm i.d. C18 with trimethylsilyl [TMS] end-capping, 110 Å, 100 × 2 mm) and a guard column (Phenomenex Inc, Torrance, CA, USA). The flow rate was set at 0.3 mL/min for mobile phase A (water with 0.1% formic acid) and mobile phase B (acetonitrile with 0.1% formic acid). Both phases were mixed in the gradient program: 5 min, 0% B; 20 min, 66% B; 35 min, 95% B. The stop time was set at 35 min. The injection volume was 10 µL (15). Mass spectrometry data acquisition was performed on Agilent 6530B QTOF Accurate-Mass QTOF spectrometer working with Dual Agilent Jet Stream spray source (ESI) (Agilent Technologies, Santa Clara, CA, USA) operating in the positive ion mode. The acquisition was performed in a scan range from 100 to 3,000 m/z, 2 spectra/s, in the collision energy of 10 and 30 eV. The drying gas temp. and flow were 350°C and 12 L/min, respectively; sheath gas temp. and flow were 400°C and 12 L/min, respectively; the nebulizer pressure was 40 psig, the capillary voltage was 4,000 V, and the skimmer was 65 V. For accurate online mass calibration, the standard masses (121.0508 and 922.0097) were injected directly into the ion source (15). Mass Hunter Qualitative Analysis software (version B.07.00; Agilent Technologies, Santa Clara, CA, USA) was used to process raw data. The mzDATA files generated in Mass Hunter Qualitative Analysis (version B.07.00; Agilent Technologies, Santa Clara, CA, USA) were subjected to feature detection in open-source software XCMS (version 3.7.1, https://xcmsonline.scripps.edu). The CentWave algorithm was applied for normalization, scaling, and filtering before pairwise statistical analysis. Signal intensity 500 was set as a threshold for mass traces retained only if present in at least five replicates. The correction of retention time was done in the obiwarp method. A sample containing only usnic acid and a sample of culture medium without bacteria served as background. Lipids were annotated in MS-LAMP software (http://ms-lamp.igib.res.in) with an integrated *M. tuberculosis* Lipidome database. The m/z values of features detected in XCMS were assigned to singly protonated ions (M + H)^+^ or adducts (M + Na)^+^ with allowed 0.05 m/z mass difference.

### Statistical analysis

The PLS-DA was performed using SIMCA-P 16.0.2 (Umetrics, Umeå, Sweden) to distinguish and define the differences in lipid profile between treated and control bacteria samples. The category-based (treated and control) regression model was established, and key variables affecting the grouping were determined. Score plots were used to visualize the separation between classes. Loading plots were examined to understand the contribution of each variable to the components. VIP enabled the identification of the most influential variables. Variables with VIP scores greater than 1.0 were considered significant. *R*² and *Q*² values for each component were used to explain the variance and predictive variance of the model.

### Conclusions

The findings from the metabolomic and transcriptomic analyses of *M. tuberculosis* under the influence of usnic acid highlight several key mechanisms by which this agent affects bacterial cells, primarily targeting metabolic processes, cell envelope integrity, and iron metabolism. Usnic acid-induced significant downregulation of glycerolipids, glycerophospholipids, and prenol lipids, suggesting a general reduction in cellular metabolic activity. The lowered levels of DIMA and DIMB, crucial for maintaining cell wall impermeability, and the downregulation of the mas gene, which is essential for dimycocerosyl phthiocerol biosynthesis, further indicate that the drug interferes with cell wall synthesis, potentially compromising the integrity of the bacterial envelope. The increase in mycobactins, which are iron-binding molecules, points to the importance of iron in the stress response triggered by usnic acid. The redirection of iron toward the synthesis of Fe-S clusters and their involvement in alternative energy production pathways, such as cytochrome P450, reveals how *M. tuberculosis* shifts its energy production in response to oxidative stress. This shift, also reflected by the downregulation of key TCA cycle enzymes, shows the bacteria’s reliance on alternative electron transport systems when oxidative phosphorylation is disrupted. The overexpression of the fprB gene, coding for ferredoxin reductase, and the involvement of cytochrome P450 pathways indicate that mycobacteria activated alternative electron transport mechanisms to maintain energy production under stress. This phenomenon aligns with previous reports of *M. tuberculosis* switching to cytochrome P450 during dormancy or under other antibiotic stresses.

The contrasting responses between the virulent H37Rv strain and the avirulent H37Ra strain suggest that usnic acid affects *M. tuberculosis* strains differently depending on their susceptibility. The more susceptible H37Rv strain showed metabolic suppression, including diminished lipid synthesis, while the less susceptible H37Ra strain exhibited increased molecules linked to redox balance and cell envelope rearrangements. This difference could be attributed to the distinct MIC values for usnic acid between these strains.

These findings suggest that usnic acid’s antimicrobial activity against *M. tuberculosis* involves a multifaceted approach, targeting lipid metabolism, cell wall synthesis, iron metabolism, and energy production. The differences in the responses of virulent and avirulent strains further underscore the complexity of mycobacterial resistance mechanisms and the potential of usnic acid as a lead compound for future therapeutic development.

## Data Availability

Metabolomic and transcriptomic data are available from Zenodo at https://doi.org/10.5281/zenodo.13788854.
